# Effects of PPARγ ligands on TGF-β1-induced epithelial-mesenchymal transition in alveolar epithelial cells

**DOI:** 10.1186/1465-9921-11-21

**Published:** 2010-02-23

**Authors:** Xiahui Tan, Hayat Dagher, Craig A Hutton, Jane E Bourke

**Affiliations:** 1 Department of Pharmacology, University of Melbourne, Victoria, Australia; 2 School of Chemistry and Bio21 Institute of Molecular Science and Biotechnology, University of Melbourne, Victoria, Australia

## Abstract

**Background:**

Transforming growth factor β1 (TGF-β1)-mediated epithelial mesenchymal transition (EMT) of alveolar epithelial cells (AEC) may contribute to lung fibrosis. Since PPARγ ligands have been shown to inhibit fibroblast activation by TGF-β1, we assessed the ability of the thiazolidinediones rosiglitazone (RGZ) and ciglitazone (CGZ) to regulate TGF-β1-mediated EMT of A549 cells, assessing changes in cell morphology, and expression of cell adhesion molecules E-cadherin (epithelial cell marker) and N-cadherin (mesenchymal cell marker), and collagen 1α1 (COL1A1), CTGF and MMP-2 mRNA.

**Methods:**

Serum-deprived A549 cells (human AEC cell line) were pre-incubated with RGZ and CGZ (1 - 30 μM) in the absence or presence of the PPARγ antagonist GW9662 (10 μM) before TGFβ-1 (0.075-7.5 ng/ml) treatment for up to 72 hrs. Changes in E-cadherin, N-cadherin and phosphorylated Smad2 and Smad3 levels were analysed by Western blot, and changes in mRNA levels including COL1A1 assessed by RT-PCR.

**Results:**

TGFβ-1 (2.5 ng/ml)-induced reductions in E-cadherin expression were associated with a loss of epithelial morphology and cell-cell contact. Concomitant increases in N-cadherin, MMP-2, CTGF and COL1A1 were evident in predominantly elongated fibroblast-like cells. Neither RGZ nor CGZ prevented TGFβ1-induced changes in cell morphology, and PPARγ-dependent inhibitory effects of both ligands on changes in E-cadherin were only evident at submaximal TGF-β1 (0.25 ng/ml). However, both RGZ and CGZ inhibited the marked elevation of N-cadherin and COL1A1 induced by TGF-β1 (2.5 ng/ml), with effects on COL1A1 prevented by GW9662. Phosphorylation of Smad2 and Smad3 by TGF-β1 was not inhibited by RGZ or CGZ.

**Conclusions:**

RGZ and CGZ inhibited profibrotic changes in TGF-β1-stimulated A549 cells independently of inhibition of Smad phosphorylation. Their inhibitory effects on changes in collagen I and E-cadherin, but not N-cadherin or CTGF, appeared to be PPARγ-dependent. Further studies are required to unravel additional mechanisms of inhibition of TGF-β1 signalling by thiazolidinediones and their implications for the contribution of EMT to lung fibrosis.

## Background

In idiopathic pulmonary fibrosis (IPF), the most prominent change in lung architecture is the appearance of fibroblast foci in the lung interstitium. Diminution of the alveolar epithelial lining is accompanied by accumulation of fibroblast-like mesenchymal cells that secrete excessive extracellular matrix proteins such as fibrillar collagen I and III [1]. Current treatment for IPF includes glucocorticoids, which show poor efficacy and do not prevent disease progression or reduce the high mortality rates within 5 years of diagnosis [1-3]. It is critical therefore to identify novel therapeutic agents that target foci development to address this area of pressing medical need.

The exact origin of increased fibroblast-like cells within foci is not known but these cells possess myofibroblast-like properties evidenced by expression of α-smooth muscle actin (αSMA) [4,5]. In IPF, myofibroblasts are regarded as the main perpetrators of fibrosis as they appear to be the major source of ECM proteins such as fibrillar collagen I [6,7]. Initially, it was thought that myofibroblasts develop from the differentiation of resident parenchymal fibroblasts in response to profibrotic cytokines, such as transforming growth factor-β1 (TGF-β1) [8-11]. Recently however, immunostaining of lung biopsies from IPF patients has revealed fibroblast-like cells expressing the surfactant protein C (SP-C) normally synthesised and secreted by type II alveolar epithelial cells (AECII) [4]. This suggests that in addition to regenerating damaged alveolar epithelial lining [12], AECII may undergo epithelial-mesenchymal transition (EMT) to contribute to foci development in the disease context.

TGF-β1 has been identified as a potent stimulus for EMT, whereby epithelial cells acquire hyperplasticity and develop a mesenchymal-cell phenotype [5,13-15]. TGF-β1 treatment has been shown to alter the cell morphology of the human AECII derived A549 cell line from cobblestone-shaped to a fibroblastoid appearance [13,15]. Phenotypic markers associated with EMT included diminished expression of E-cadherin, a cell anchoring protein expressed specifically by epithelial cells, and elevated expression of N-cadherin, normally present at relatively higher levels in fibroblasts [8,10]. These alterations were accompanied by increased secretion of the gelatinase matrix metalloproteinase-2 (MMP-2) [13,14], increased cell motility [15,16] and *de novo *synthesis of fibrillar collagen I and III [13]. Given the established actions of TGF-β1 on fibroblast differentiation, EMT and collagen synthesis, studies have investigated potential therapeutic benefits of targeting profibrotic effects of TGF-β1.

Peroxisome proliferator-activated receptor γ (PPARγ) is a nuclear hormone receptor activated by the thiazolidinedione class of anti-diabetic drugs that may have a role in the regulation of both inflammation and fibrosis in the lung [17,18]. In cultured lung fibroblasts from subjects with and without IPF, the PPARγ ligands rosiglitazone (RGZ), troglitazone (TGZ) and ciglitazone (CGZ) prevented TGF-β1-mediated increases in αSMA expression and fibrillar collagen synthesis [8,9]. Similar findings have been observed in skin fibroblasts [19], with PPARγ ligands inhibiting TGF-β1 signalling via the Smad pathway. Additional *in vivo *studies revealed that treatment with TGZ and CGZ protected against bleomycin-induced lung fibrosis in mice [9], a model in which glucocorticoids are ineffective [20].

To date, the ability of PPARγ ligands to antagonize TGF-β1-mediated changes associated with EMT in human AECII has not been explored. In the current study, we use a well-validated model of EMT in the A549 human alveolar cell line in which a suite of morphological, phenotypic and functional markers and outcomes have been well characterised [13]. Our results demonstrate that RGZ and CGZ inhibit several TGF-β1-induced changes in markers of EMT and lung fibrosis, including cadherin proteins and collagen gene expression, to provide further support for the antifibrotic potential of the thiazolidinedione class of compounds.

## Methods

### Materials

Recombinant human TGF-β1 was purchased from R&D systems (Minneapolis, MN). RGZ, CGZ and GW9662 and rabbit polyclonal antibody against human PPARγ were from Cayman Chemical Corporation (Ann Arbor, MI) and sheep polyclonal Texas-red conjugated anti-rabbit antibody was from Abcam (Cambridge, UK). Mouse monoclonal antibodies against human E-cadherin and N-cadherin were from BD Transduction Laboratory (Oxford, UK), against human β-actin from Abcam (Cambridge, UK) and against human αSMA from Sigma Aldrich (St. Louis, MO). Rabbit monoclonal antibodies against human Smad2 and Smad3, and phosphorylated Smad2 and Smad3 were from Cell Signaling Technology (Beverly, MA). Sheep HRP-conjugated polyclonal anti-mouse and anti-rabbit antibodies were from Chemicon International (Temecula, CA). Quantitect primers for the human genes COL1A1, COL3A1 and 18s rRNA were from Qiagen (Hilden, Germany) and the primer sequences for αSMA (ACTA2) and MMP-2 were previously reported in [14] and were synthesised by Geneworks (Adelaide, Australia). Phosphate buffered saline (PBS) was from Oxoid (Basingstoke, UK), with all other chemicals and reagents from Sigma Aldrich.

### Cell Culture and Drug Treatment

Human type II alveolar epithelial cell line A549 (ATCC, Manassas, VA) was maintained in high glucose-DMEM (Invitrogen, Carlsbad, CA) containing 10% (v/v) FBS, 15 mM HEPES, 20 mM sodium bicarbonate, and 2 mM L-glutamine at 37°C in a humidified 5% CO_2 _atmosphere. Cells were seeded at a density of 5 × 10^4 ^cells per well onto 6-well plates, or at 2 × 10^3 ^cells per well onto 8-well Lab-Tek Chamber slides which were pre-coated with 0.1 mg/ml (w/v) poly-L-lysine solution. After overnight attachment, cells were maintained in serum-free DMEM containing 0.25% (w/v) bovine serum albumin (BSA) for 24 hr.

PPARγ ligands were prepared as 30 mM stocks in dimethylsulfoxide (DMSO), with 0.1% DMSO used as vehicle control for treatment of cells. Cells were preincubated with PPARγ agonists RGZ or CGZ (1-30 μM) in the absence or presence of the PPARγ antagonist GW9662 (10 μM) [21] for 60 min prior to stimulation with TGF-β1 (0.75-7.5 ng/ml) for 2-24 hr for immunocytochemical studies, 24 hr for mRNA analysis or up to 72 hr for protein analysis. These drugs did not affect A549 cell viability as measured by Trypan blue staining. The concentration of GW9662 used has been shown to have no effect on basal PPRE promoter activity but to suppress RGZ-induced activation of the PPRE reporter [22,23] and the antiproliferative effects of RGZ in human cultured airway smooth muscle [24].

### Immunocytochemistry

Cells on chamber slides were washed with PBS, fixed in ice-cold methanol for 10 min, and then blocked with 2% BSA in PBS for 1 hr at room temperature. Cells were then incubated with primary PPARγ antibody (1:100) for 2 hr at room temperature. After washing, antibody binding was detected by incubating with sheep polyclonal Texas-red conjugated anti-rabbit antibody (1:100) for 1 hr. Cell nuclei were visualised by staining with 1 μg/ml (w/v) Hoescht-33258 dye for 20 min at room temperature. Images were collected with a Carl Zeiss Axioscope microscope system at 20× magnification.

### Preparation of cytoplasmic and nuclear cell extracts

A549 cells were washed with PBS, then treated with whole cell lysis buffer (100 mM NaCl, 0.5% w/v 10 mM Tris-HCl (pH = 7.5), 0.5% w/v sodium deoxycholate, 2 mM sodium EDTA and 1% v/v Triton-X) supplemented with 1% v/v protease inhibitor cocktail and phosphatase inhibitor cocktail 2. Cells were lysed for 20 min on ice before centrifugation and collection of supernatants.

To separate cytosolic and nuclear proteins, cells were treated for 10 min on ice with cytosolic lysis buffer (10 mM HEPES, 10 mM KCl, 10 mM EDTA, 1 mM DTT) supplemented with 1% v/v protease inhibitor cocktail and 0.4% v/v IGEPAL. After centrifugation, the supernatant containing the cytosolic fraction was collected and the remaining pellet treated with lysis buffer for cell nuclei (10 mM HEPES, 400 mM NaCl, 1 mM EDTA, 10% v/v glycerol, pH 7.9) supplemented with 1% v/v protease inhibitor cocktail and 1 mM dithiothreitol. Samples were homogenised by vortexing and then incubated at 200 rpm on ice for 2 h on a rocking platform.

All protein supernatant samples were collected following centrifugation at 13,000 g (5 min, 4°C) and stored at -20°C. Total protein concentration was measured via a spectrophotometer (Thermoplus, Finland) using the bicinchoninic acid (BCA) protein assay kit (Bio-Rad, Hercules, CA) with BSA utilised as the protein standard.

### SDS-PAGE and Western blot

20 μg of total protein from each sample was separated on 10% polyacrylamide gels (Bio-Rad). After electrophoresis, separated proteins were transferred onto Hybond Nitrocellulose membranes (Amersham, Buckinghamshire, UK). Membranes were then blocked for 1 hr at room temperature with 5% w/v skimmed milk solution or 2% w/v BSA in TBST. Membranes were then probed with primary antibodies (1:10,000 for β-actin, 1:1000 for other antibodies) for 18 hr at 4°C. After washing with TBST, membranes were probed with either sheep anti-mouse or anti-rabbit HRP-conjugated secondary antibodies for 1 hr at room temperature. Immunoblots were visualised by enhanced chemiluminescence (GE Healthcare, Chalfont St Giles, UK) and band densities quantified using ImageQuant TL software after image acquisition with an ImageQuant 350 (GE Healthcare). Results were expressed relative to β-actin band density used as a loading control.

### Real Time Quantitative Reverse Transcription-PCR

Total RNA was extracted using the RNeasy Kit (Qiagen). For complementary DNA (cDNA) synthesis 1 μg of total RNA was reverse transcribed using Superscript III (Invitrogen). cDNA equivalent to 10 ng of total RNA was used for all PCR reactions in the presence of 100 nM of forward and reverse primers (refer to Materials section). All PCR reactions were performed using Platinum SYBR Green qPCR SuperMix-UDG (Invitrogen) in an ABI Prism 7900 HT sequence detection system (Applied Biosystems, Foster City, CA). For normalization of all RT-PCR data, 18s rRNA expression was used as a reference gene. Relative transcript abundance of COL1A1, MMP-2 and CTGF were expressed in ΔCt values (ΔCt = Ct^reference^-Ct^target^). Relative fold changes in transcript levels compared to basal levels were calculated as 2^ΔΔCt ^(ΔΔCt = ΔCt^treatment^-ΔCt^basal^).

### Statistical Analysis

Data were expressed as mean response ± S.E.M. for n repeated experiments and analysed using Graph Pad Prism version 5.0 software. The effects of TGF-β1 on cadherin expression were analysed by one-way ANOVA on raw densitometric data normalised for β-actin as loading control. The effects of PPARγ ligands in the presence and absence of antagonist on TGF-β1-mediated changes of EMT markers were tested by one-way repeated measures ANOVA followed by Bonferroni post hoc test for multiple comparisons with data normalised to control levels in the absence of TGF-β1. Effects were considered to be statistically significant when P < 0.05.

## Results

### PPARγ expression in A549 cells

**Figure 1:**
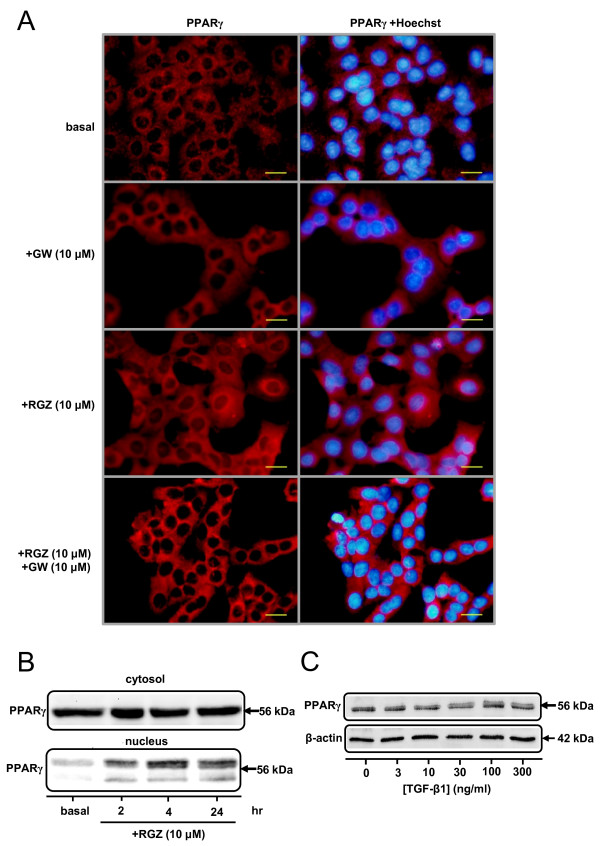
**PPARγ expression and localization in A549 cells**. (A) Immunocytochemistry for PPARγ in A549 cells. Cells were grown to confluence on chamber slides, serum deprived for 24 hr and then treated with either vehicle (0.1% DMSO, left) for basal, 10 μM GW9662, 10 μM RGZ or 10 μM RGZ + 10 μM GW9662 for 24 hr. PPARγ localization in the cells was detected by immunocytochemistry (left column) and the nuclei visualised by Hoechst staining (right column). (B) Western blot for PPARγ expression in the cytosol and nuclei of serum-deprived A549 cells in the absence and presence of RGZ (10 μM) for 2 to 24 hr. Basal levels were measured at 2 hr after vehicle (0.1% DMSO) treatment. (C) Western blot for PPARγ expression in A549 cells in the absence and presence of TGF-β1 (72 hrs). All images and blots are representative of 3 separate experiments. In the ICC images bar = 100 μm.

Immunocytochemical staining for PPARγ was performed in serum-deprived A549 cells. Under basal conditions, PPARγ was detected in both cytoplasm and nuclei, with higher levels of expression evident in the cytosol (Figure 1A). However, in cells treated with the PPARγ ligand RGZ, both perinuclear and nuclear staining for PPARγ was increased within 2 hr with maximal effects observed at 24 hr (Figure 1A). Changes in receptor localization in the presence of RGZ were prevented by the PPARγ antagonist GW9662 (Figure 1A). RGZ-induced nuclear translocation was confirmed by Western blotting showing increased levels of PPARγ in nuclear extracts at 2, 4 and 24 hrs (Figure 1B). Cellular PPARγ protein levels were not regulated by treatment with TGF-β1 (Figure 1C).

Having verified PPARγ receptor expression and the ability of RGZ to cause receptor translocation in these cells, we next investigated whether thiazolidinediones could regulate TGF-β1-mediated changes in markers of EMT.

### Thiazolidinediones do not regulate basal E-cadherin and N-cadherin expression

**Figure 2:**
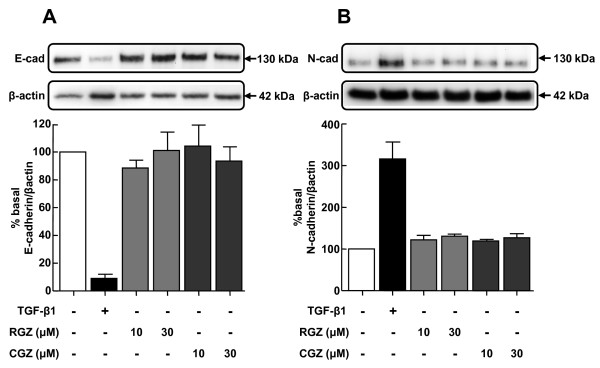
**Regulation of basal E-cadherin and N-cadherin expression by PPARγ ligands**. (A) Effect of TGF-β1 or PPARγ ligands on expression of epithelial marker E-cad (n = 4). (B) Effect of TGF-β1 or PPARγ ligands on expression of mesenchymal marker N-cad (n = 4). Cell lysates were prepared from A549 cells pre-incubated with vehicle, TGF-β1 (2.5 ng/ml), or RGZ or CGZ at the concentrations indicated for 72 hr. Densitometric analysis values for band intensities from each Western blot were normalised to β-actin and expressed as a percentage of basal levels. Each point represents mean ± s.e.m.

Under basal conditions, A549 cells expressed relatively higher levels of E-cadherin than N-cadherin, consistent with their epithelial phenotype. Treatment with RGZ and CGZ at concentrations up to 30 μM did not affect basal levels of either cadherin (Figure 2A, B).

### Thiazolidinediones inhibit TGF-β1-induced changes in E-cadherin expression via PPARγ

**Figure 3:**
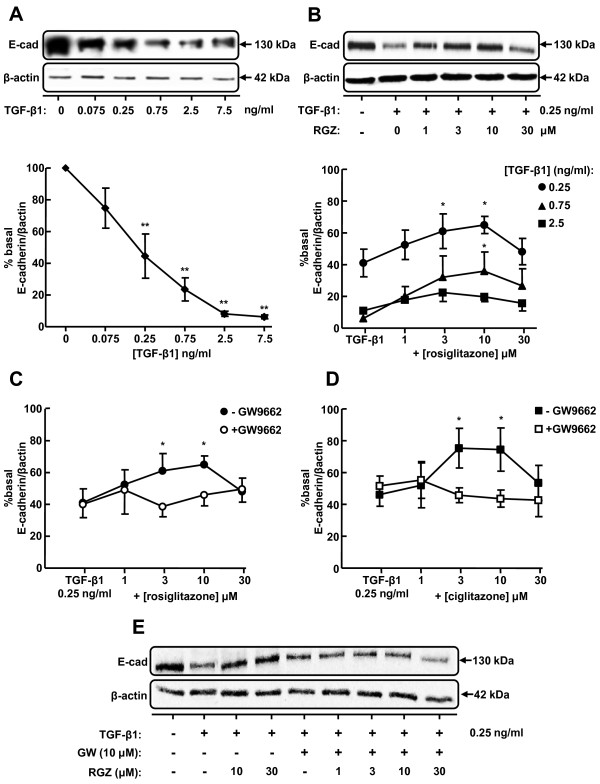
**Regulation of TGF-β1-induced E-cadherin expression by PPARγ ligands**. (A) Effect of TGF-β1 on expression of epithelial marker E-cad (n = 4). (B) Effect of RGZ on regulation of E-cad expression by TGF-β1 (n = 6). (C, D) Effect of the PPARγ antagonist GW9662 (10 μM) on regulation of TGF-β1-mediated E-cad expression by RGZ (n = 4) and CGZ (n = 4). (E) Western blot showing the effect of GW9662 on RGZ-mediated inhibition of TGF-β1 effect on E-cad expression (representative of n = 4 experiments). Cell lysates were prepared from A549 cells pre-incubated with vehicle, RGZ or CGZ with or without GW9662 for 1 hr, stimulated with TGF-β1 at the concentrations indicated for 72 hr. Densitometric analysis values for band intensities from each Western blot were normalised to β-actin and expressed as a percentage of basal levels. Each point represents mean ± s.e.m. * P < 0.05, compared with basal (A) TGF-β1 (B-D).

TGF-β1 treatment of A549 cells for 72 hr reduced basal expression of E-cadherin in a concentration-dependent manner (Figure 3A). The 60% reduction in E-cadherin levels in the presence of 0.25 ng/ml TGF-β1 was partially inhibited by both RGZ and CGZ at concentrations up to 10 μM but this effect was not maintained at 30 μM (Figure 3B, D). The effectiveness of RGZ was also reduced when cells were stimulated with 0.75 or 2.5 ng/ml TGF-β1 (Figure 3B), and CGZ was unable to prevent the maximal decrease in E-cadherin at these TGF-β1 concentrations (data not shown). The PPARγ antagonist GW9662 alone did not affect the reduction of E-cadherin expression induced by 0.25 ng/ml TGF-β1 but prevented the partial inhibitory effects of both PPARγ ligands (Figure 3C, D, E).

### Thiazolidinediones inhibit TGF-β1-induced changes in N-cadherin expression independent of PPARγ

**Figure 4:**
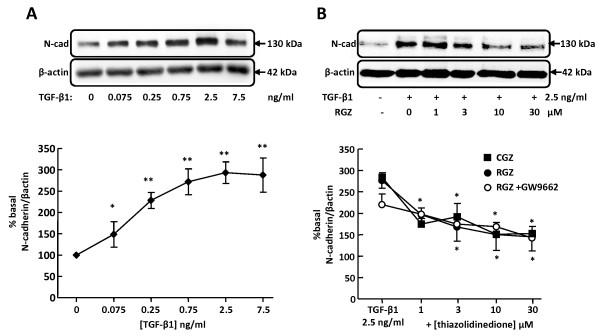
**Regulation of TGF-β1-induced N-cadherin expression by PPARγ ligands**. (A) Effect of TGF-β1 on expression of mesenchymal marker N-cad (n = 4). (B) Effect of PPARγ ligands on regulation of N-cad expression by 2.5 ng/ml TGF-β1 (RGZ, n = 6; RGZ + GW9662, n = 6; CGZ, n = 6). Cell lysates were prepared from A549 cells pre-incubated with vehicle, RGZ or CGZ with or without GW9662 for 1 hr, stimulated with TGF-β1 at the concentrations indicated for 72 hr. Densitometric analysis values for band intensities from each Western blot were normalised to β-actin and expressed as a percentage of basal levels. Each point represents mean ± S.E.M. * P < 0.05, ** P < 0.01 compared  compared with basal (A) or TGF-β1 (B).

Low basal expression of N-cadherin in A549 cells was increased approximately 3-fold by TGF-β1 (Figure 4A). In contrast, basal expression of αSMA was not affected by TGF-β1 at concentrations up to 7.5 ng/ml (data not shown). Both RGZ and CGZ partially inhibited the maximum increase in N-cadherin expression in response to 2.5 ng/ml TGF-β1 (Figure 4B). The inhibitory effects of RGZ and CGZ were maintained in the presence of GW9662 (RGZ Figure 4B, CGZ data not shown).

### Thiazolidinediones inhibit TGF-β1-induced changes in collagen I and CTGF mRNA

**Figure 5:**
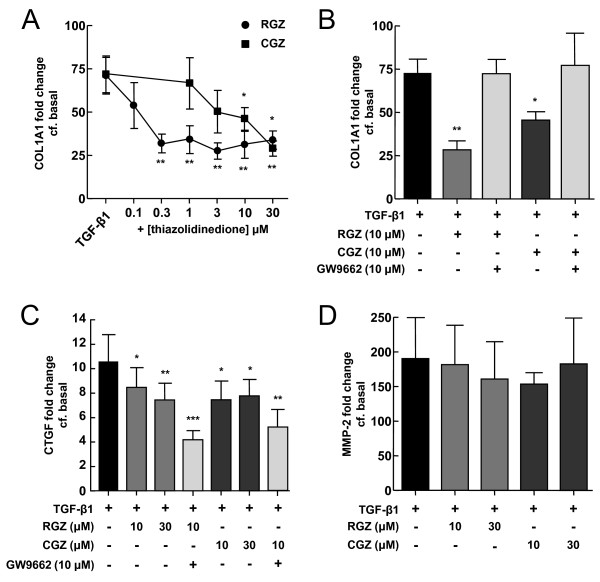
**Regulation of TGF-β1-induced COL1A1, CTGF and MMP-2 mRNA levels by PPARγ ligands**. (A) Effect of PPARγ agonists on regulation of COL1A1 mRNA levels by TGF-β1 (n = 5). (B) Effect of the PPARγ antagonist GW9662 (10 μM) on regulation of TGF-β1-mediated COL1A1 mRNA by RGZ (n = 6) and CGZ (n = 4). (C) and (D) Effect of PPARγ ligands on regulation of CTGF and MMP-2 mRNA levels by TGF-β1 (n = 4,7). Total RNA was collected from A549 cells pre-incubated with vehicle, RGZ or CGZ with or without GW9662 for 1 hr and then stimulated with 2.5 ng/ml TGF-β1 for 24 hr. Results were normalised to 18s rRNA levels and expressed as fold change from basal levels. Each point represents mean ± S.E.M. * P < 0.05, ** P < 0.01, *** P < 0.001 compared with TGF-β1.

Treatment of A549 cells with 2.5 ng/ml TGF-β1 for 24 hr caused 71 ± 11 fold (n = 5, P < 0.01) and 190 ± 59 fold (n = 4, P < 0.01) increases in COL1A1 and MMP-2 mRNA levels respectively (Figure 5A, D). The increase in CTGF mRNA level was more modest (11 ± 2 fold, n = 7, P < 0.05, Figure 5C). Basal levels of collagen III or αSMA mRNA were not significantly upregulated by TGF-β1 (data not shown).

The TGF-β1-induced increase in COL1A1 mRNA was attenuated by both RGZ and CGZ to a similar extent, although RGZ was markedly more potent (Figure 5A). Both RGZ and CGZ partially inhibited the increase in CTGF (Figure 5C). The effects of both PPARγ ligands on COL1A1 but not CTGF mRNA were abolished in the presence of GW9662 (Figure 5B, C). In contrast, the increase in MMP-2 mRNA following TGF-β1 treatment was not inhibited by RGZ or CGZ (Figure 5D, n = 7, P < 0.05).

### Thiazolidinediones do not prevent TGF-β1-induced changes in cell morphology

**Figure 6:**
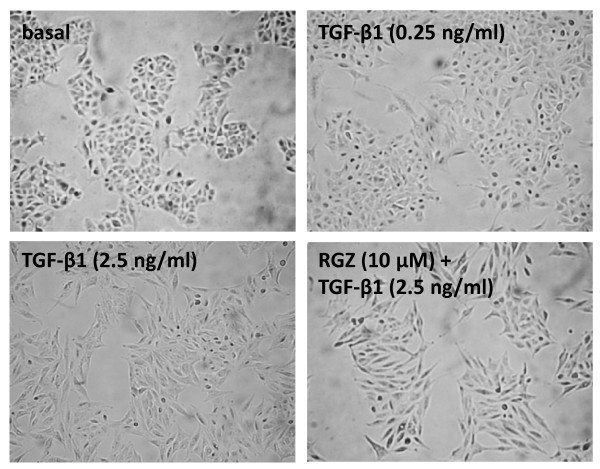
**Regulation of TGF-β1-induced changes in cell morphology by rosiglitazone**. A549 cells were incubated with vehicle (0.1% DMSO), TGF-β1 (0.25 ng/ml), TGF-β1 (2.5 ng/ml) or TGF-β1 (2.5 ng/ml) + RGZ (10 μM) for 72 hr and photographed at 100× magnification. The images are representative of 4 separate experiments.

In the presence of 2.5 ng/ml TGF-β1, the morphology of A549 cells changed from the classical cobblestone appearance of alveolar epithelial cells to predominantly elongated fibroblast-like cells (Figure 6). These changes were not evident following treatment with 0.25 ng/ml TGF-β1. Pre-treatment with RGZ or CGZ did not affect cell morphology in the absence or presence of TGF-β1 (Figure 6, CGZ not shown).

### Thiazolidinediones do not affect TGF-β1-mediated phosphorylation of Smad2 and Smad3

**Figure 7:**
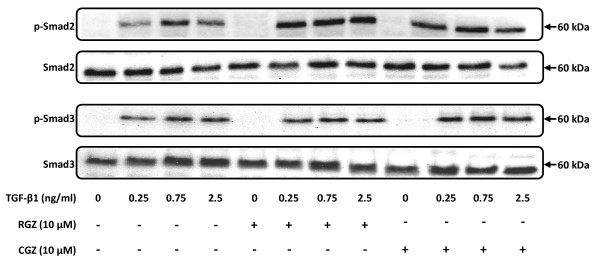
**Regulation of TGF-β1-induced phosphorylation of Smad2 and Smad3 by PPARγ ligands**. Cell lysates were prepared from A549 cells pre-treated with vehicle, RGZ (10 μM) or CGZ (10 μM) for 1 hr and then treated with TGF-β1 at the indicated concentrations for 1 hr. The Western blot is representative of 3 separate experiments.

Under basal conditions in the absence of TGF-β1, A549 cells expressed Smad2 and Smad3 proteins, and treatment with RGZ or CGZ had no effect (Figure 7). The phosphorylated forms of these proteins could be detected as early as 15 min after TGF-β1 stimulation (data not shown), with maximum phosphorylation after 1 hr. Pretreatment with RGZ or CGZ did not prevent the increase in levels of phosphorylated Smad proteins in response to TGF-β1 (Figure 7).

## Discussion

In this study, we show that the PPARγ ligands RGZ and CGZ inhibit TGF-β1-induced changes in E-cadherin, N-cadherin, collagen I and CTGF expression in A549 cells in the absence of regulatory effects on cell morphology. RGZ was consistently more effective than CGZ in inhibiting PPARγ dependent changes in markers of EMT, in agreement with their relative binding affinities for PPARγ. However, since inhibitory effects occurred via both PPARγ-dependent and PPARγ-independent pathways and were not associated with inhibition of Smad phosphorylation, it is proposed that multiple mechanisms underlie potential antifibrotic actions of PPARγ ligands in these cells.

Recent studies provide evidence that TGF-β1-induced EMT of AECII may contribute to the *de novo *appearance of myofibroblasts in fibrotic lungs [4,5,25-27]. Although these findings are not universal [28], it has been shown that induction of lung fibrosis in mice by overexpression of active TGF-β1 or bleomycin treatment resulted in the accumulation of mesenchymal cells with AECII origins adjacent to fibrotic lesions [4,25]. Additionally, fibroblast-like cells in lung biopsies from IPF patients expressed the AECII surfactant protein SP-C [4].

To explore potential regulation of EMT in AECII by PPARγ ligands, we have used A549 cells as a model of human AECII. A549 cells possess many features of normal AECII cells [29] and have been used in numerous studies examining EMT [13-16,30-32]. We characterised EMT in A549 cells by detecting changes in cell morphology, E-cadherin and N-cadherin levels, and collagen I, CTGF and MMP-2 gene expression. These markers facilitate identification of cells along the spectrum of transition from epithelial to mesenchymal phenotype. Critically, A549 cells express PPARγ, the receptor target of the thiazolidinedione class of drugs which includes RGZ and CGZ. In the current study, nuclear translocation of PPARγ by RGZ provided evidence that PPARγ activation could potentially regulate cellular functions of A549 cells, including EMT. This translocation was prevented by the PPARγ antagonist GW9662, supporting the use of this pharmacological tool to explore the PPARγ-dependence of the actions of RGZ and CGZ.

In this study, treatment of cells with RGZ or CGZ in the absence of TGF-β1 failed to elicit any detectable changes in expression of cell adhesion molecules or morphology. Direct regulation of EMT by PPARγ ligands may vary depending on cellular origin, since PPARγ activation by RGZ has previously been shown to promote EMT of gastrointestinal epithelial cells, characterised by increased cell scattering and altered cell morphology [33].

Consistent with previous findings in A549 cells, TGF-β1 treatment caused cells to lose their polygonal appearance and cell-cell contacts leading to the acquisition of elongated, spindle-shaped morphology consistent with fibroblasts [13]. TGF-β1 also altered the expression of cell adhesion molecules consistent with EMT as reported previously [13,30]. Significant reductions in E-cadherin, a protein expressed only by epithelial cells and diminished during EMT [26], were evident even in the absence of obvious morphological changes, suggesting that a loss of >60% E-cadherin is required before cell morphology is altered. Under these conditions, the partial inhibition of the reduction in E-cadherin by both RGZ and CGZ was PPARγ dependent, since it was abolished in the presence of the selective PPARγ antagonist GW9662. The modest inhibitory effect was not maintained at the highest concentrations of PPARγ ligands tested, but the mechanism for this loss of activity was not explored.

The maximum effect of TGF-β1 elicited was a 90% loss of E-cadherin expression, suggesting that cells that have undergone EMT may still retain epithelial phenotypic markers. *In vivo *studies have previously reported retention of the AECII specific SP-C and pro SP-B proteins in mesenchymal cells derived from EMT [4,5]. Thiazolidinedione treatment was unable to prevent this maximal reduction in E-cadherin expression accompanied by loss of epithelial morphology.

Low expression of N-cadherin and αSMA were detected under basal conditions in A549 cells, despite the classification of these markers as mesenchymal specific [34-36]. Similar findings have been described in both A549 cells [13,31], and in RLE-6TN cells [37], a rat AECII cell line reported to undergo EMT upon TGF-β1 treatment [5,37,38]. In RLE-6TN cells, basal expression of αSMA was attributed to constitutive activation of TGF-β1 type I receptor kinase (TGFβRI) [37], and may also contribute to basal N-cadherin and αSMA expression in A549 cells.

Following TGF-β1 treatment, concomitant increases in N-cadherin accompanied reductions in E-cadherin expression in A549 cells. Although both proteins mediate cell to cell attachment, cell adhesion by E-cadherin is four times stronger than adhesion by N-cadherin [39]. In squamous carcinoma cells, N-cadherin has also been shown to promote scattering and increased motility [40]. It is likely then that the relative expression levels of these molecules contributed to the loss of cell-cell contact in A549 cells evident at higher concentrations of TGF-β1.

The maximum TGFβ1-induced increase in N-cadherin expression was approximately 3-fold higher than basal levels. In contrast to their limited effects on the reduction in E-cadherin, both RGZ and CGZ were able to decrease the elevation in N-cadherin levels to a similar extent following treatment with 2.5 ng/ml TGF-β1. In addition, the inhibitory effects of PPARγ ligands on the increased N-cadherin expression were not attenuated by GW9662, suggesting a separate PPARγ-independent pathway.

In addition to altered cadherin expression, A549 cells stimulated with TGF-β1 displayed other features similar to lung fibroblasts. Under basal conditions, A549 cells do not synthesize fibrillar collagen I or CTGF, and only express low levels of MMP-2 [13,14,41]. MMP-2 activity is thought to be an important contributor to EMT by facilitating basement membrane breakdown and migration of cells into the interstitium [42-44]. CTGF expression is induced by TGF-β1 and has profibrotic properties including stimulation of fibrillar collagen production and myofibroblast accumulation [41,45]. As confirmed in this study, stimulation with TGF-β1 caused marked increases in mRNA for COL1A1, which encodes the α1 chain of mature collagen I fibers, as well as MMP-2 and CTGF. However, in contrast to a previous report [13], significant increases in COL3A1 mRNA were not detected.

Both RGZ and CGZ significantly reduced the induction of COL1A1 and CTGF, but not MMP-2 mRNA levels by TGF-β1 in A549 cells. The higher potency of RGZ relative to CGZ to reduce COL1A1 was consistent with their relative binding affinities for PPARγ [46,47], with PPARγ dependence further supported by the abolition of their inhibitory effects in the presence of GW9662. These findings are in agreement with studies in skin and lung fibroblasts where PPARγ ligands inhibited fibrillar collagen I synthesis [8,9,19]. In this study on A549 cells, the PPARγ-dependent antifibrotic effects of sub-micromolar concentrations of RGZ are of particular interest in the context of excessive collagen production by myofibroblasts in lung fibrosis.

Overall, the current findings suggest that the inability of PPARγ ligands to prevent changes in A549 morphology and cell-cell contact at high TGF-β1 concentrations may be due to their limited capacity to exert inhibitory effects on TGF-β1-induced changes in E-cadherin and MMP-2 expression. However, marked inhibitory effects of RGZ on collagen and N-cadherin were maintained with maximal TGF-β1 stimulation and appeared to be via PPARγ-dependent and PPARγ-independent pathways respectively. Although PPARγ ligands may not prevent the acquisition of a mesenchymal phenotype, their impact on collagen synthesis may provide protection from the progression of fibrosis. Ideally, these findings should be extrapolated to primary human alveolar epithelial cells to enable further assessment of RGZ and related compounds in regulation of TGF-β1-induced pro-fibrotic functions.

In addition to examination of PPARγ dependence, further studies were conducted to assess potential mechanisms whereby PPARγ ligands regulate TGF-β1-mediated changes associated with EMT. TGF-β1-mediated EMT in A549 cells is thought to be dependent on Smad2 and Smad3 phosphorylation [13,31,37,38], since inhibition of Smad2 expression using siRNA prevented the loss of E-cadherin expression induced by TGF-β1 treatment [13]. Similar results were evident following induction of the intracellular Smad2 and Smad3 antagonist Smad7 by hepatocyte growth factor in RLE-6TN cells [37].

Several studies have shown that PPARγ activation can directly interfere with Smad signaling by either inhibiting Smad2/3 phosphorylation or Smad2/3 nuclear translocation [48,49]. In this study, Smad phosphorylation in response to TGF-β1 was not reduced by RGZ or CGZ. Our findings may be explained by an alternative mechanism identified in fibroblasts, whereby Smad-dependent transcriptional responses were blocked by PPARγ without preventing Smad 2/3 activation [50]. In this recent study, PPARγ inhibited the interaction between activated Smad2/3 and the transcriptional coactivator and histone acetyltransferase p300 induced by TGF-β1, and the accumulation of p300 on consensus Smad-binding DNA sequences and histone H4 hyperacetylation at the COL1A2 locus [50].

Alternative mechanisms for the potent PPARγ-dependent inhibitory effects observed for collagen I include direct regulation of promoter activity by PPARγ ligand-receptor complexes. This possibility is supported by evidence that constitutive COL1A2 promoter activation in PPARγ knockout mouse embryonic fibroblasts could be normalised by recovery of PPARγ expression [47]. In addition PPARγ activation is known to attenuate the signaling of other transcription factors such as Sp1 which is essential for COL1A2 gene transcription in human glomerular mesangial cells [51,52], and up regulation of EGR-1 an early-immediate response transcription factor that is also responsible for TGF-β1-mediated fibrosis [53].

Further studies are required to address the alternative mechanisms of inhibition of Smad signaling which could contribute to PPARγ-dependent regulation of TGF-β1 responses in A549 cells. In addition, PPARγ-independent effects of thiazolidinediones described in other cell types [54,55] also remain to be explored in the context of the effects of RGZ and CGZ on changes in expression of phenotypic markers of EMT.

## Conclusion

In the current study, treatment with PPARγ ligands markedly reduced TGF-β1-induced increases in collagen I, CTGF and N-cadherin in A549 cells, with inhibitory effects on changes in E-cadherin also evident. RGZ was generally more effective than CGZ, but neither PPARγ ligand inhibited the morphological changes of these cells to become fibroblast-like in appearance. The variable effects of the PPARγ antagonist GW9662 on the inhibitory effects of RGZ and CGZ implicate both PPARγ-dependent and PPARγ-independent pathways in the regulation of TGF-β1-mediated responses. Given the lack of effective therapy to inhibit the progression of lung fibrosis and the proposed contribution of EMT to this process, these findings support further exploration of the antifibrotic properties and mechanisms of action of PPARγ ligands in human alveolar epithelial cells to clarify their potential therapeutic benefit.

## List of abbreviations

AECII: type II alveolar epithelial cells; TGF-β1: transforming growth factor-β1; TGFβRI: TGF-β type I receptor kinase; COL1A1: collagen 1 α1 chain; CTGF: connective tissue growth factor; RGZ: rosiglitazone; CGZ: ciglitazone; TGZ: troglitazone; PPARγ: peroxisome proliferator-activated receptor-γ; IPF: idiopathic pulmonary fibrosis; MMP-2: matrix metalloproteinase-2; EMT: epithelial mesenchymal transition; αSMA: α-smooth muscle actin; RT-PCR: reverse transcription-polymerase chain reaction; SP-C: surfactant protein C.

## Competing interests

The authors declare that they have no competing interests.

## Authors' contributions

XT, CH and JB conceived the study. XT and HD conducted the experiments and the data was then analysed and interpreted by XT and JB. XT prepared the draft manuscript, which was edited by HD, CH and JB. All authors read and approved the final manuscript.

## References

[B1] ATS American Thoracic Society. Idiopathic pulmonary fibrosis: diagnosis and treatment. International consensus statement. American Thoracic Society (ATS), and the European Respiratory Society (ERS) Am J Respir Crit Care Med 2000 161 2 Pt 1 646 664 10673212 10.1164/ajrccm.161.2.ats3-00

[B2] Selman M Thannickal VJ Pardo A Zisman DA Martinez FJ Lynch JP Idiopathic pulmonary fibrosis: pathogenesis and therapeutic approaches Drugs 2004 64 4 405 430 10.2165/00003495-200464040-00005 14969575

[B3] Abdelaziz MM Samman YS Wali SO Hamad MM Treatment of idiopathic pulmonary fibrosis: is there anything new? Respirology 2005 10 3 284 289 10.1111/j.1440-1843.2005.00712.x 15955138

[B4] Kim KK Kugler MC Wolters PJ Robillard L Galvez MG Brumwell AN Sheppard D Chapman HA Alveolar epithelial cell mesenchymal transition develops in vivo during pulmonary fibrosis and is regulated by the extracellular matrix Proc Natl Acad Sci USA 2006 103 35 13180 13185 10.1073/pnas.0605669103 16924102 PMC1551904

[B5] Willis BC Liebler JM Luby-Phelps K Nicholson AG Crandall ED du Bois RM Borok Z Induction of epithelial-mesenchymal transition in alveolar epithelial cells by transforming growth factor-beta1: potential role in idiopathic pulmonary fibrosis Am J Pathol 2005 166 5 1321 1332 15855634 10.1016/s0002-9440(10)62351-6 PMC1606388

[B6] Phan SH The myofibroblast in pulmonary fibrosis Chest 2002 122 6 Suppl 286S 289S 10.1378/chest.122.6_suppl.286S 12475801

[B7] Zhang K Rekhter MD Gordon D Phan SH Myofibroblasts and their role in lung collagen gene expression during pulmonary fibrosis. A combined immunohistochemical and in situ hybridization study Am J Pathol 1994 145 1 114 125 7518191 PMC1887314

[B8] Burgess HA Daugherty LE Thatcher TH Lakatos HF Ray DM Redonnet M Phipps RP Sime PJ PPARgamma agonists inhibit TGF-beta induced pulmonary myofibroblast differentiation and collagen production: implications for therapy of lung fibrosis Am J Physiol Lung Cell Mol Physiol 2005 288 6 L1146 1153 10.1152/ajplung.00383.2004 15734787

[B9] Milam JE Keshamouni VG Phan SH Hu B Gangireddy SR Hogaboam CM Standiford TJ Thannickal VJ Reddy RC PPAR-gamma agonists inhibit profibrotic phenotypes in human lung fibroblasts and bleomycin-induced pulmonary fibrosis Am J Physiol Lung Cell Mol Physiol 2008 294 5 L891 901 10.1152/ajplung.00333.2007 18162602 PMC5926773

[B10] Desmouliere A Geinoz A Gabbiani F Gabbiani G Transforming growth factor-beta 1 induces alpha-smooth muscle actin expression in granulation tissue myofibroblasts and in quiescent and growing cultured fibroblasts J Cell Biol 1993 122 1 103 111 10.1083/jcb.122.1.103 8314838 PMC2119614

[B11] Vaughan MB Howard EW Tomasek JJ Transforming growth factor-beta1 promotes the morphological and functional differentiation of the myofibroblast Exp Cell Res 2000 257 1 180 189 10.1006/excr.2000.4869 10854066

[B12] Kasper M Haroske G Alterations in the alveolar epithelium after injury leading to pulmonary fibrosis Histol Histopathol 1996 11 2 463 483 8861769

[B13] Kasai H Allen JT Mason RM Kamimura T Zhang Z TGF-beta1 induces human alveolar epithelial to mesenchymal cell transition (EMT) Respir Res 2005 6 56 10.1186/1465-9921-6-56 15946381 PMC1177991

[B14] Ranganathan P Agrawal A Bhushan R Chavalmane AK Kalathur RK Takahashi T Kondaiah P Expression profiling of genes regulated by TGF-beta: differential regulation in normal and tumour cells BMC Genomics 2007 8 98 10.1186/1471-2164-8-98 17425807 PMC1858692

[B15] Keshamouni VG Michailidis G Grasso CS Anthwal S Strahler JR Walker A Arenberg DA Reddy RC Akulapalli S Thannickal VJ Differential protein expression profiling by iTRAQ-2DLC-MS/MS of lung cancer cells undergoing epithelial-mesenchymal transition reveals a migratory/invasive phenotype J Proteome Res 2006 5 5 1143 1154 10.1021/pr050455t 16674103

[B16] Yu H Konigshoff M Jayachandran A Handley D Seeger W Kaminski N Eickelberg O Transgelin is a direct target of TGF-beta/Smad3-dependent epithelial cell migration in lung fibrosis FASEB J 2008 22 6 1778 1789 10.1096/fj.07-083857 18245174

[B17] Lakatos HF Thatcher TH Kottmann RM Garcia TM Phipps RP Sime PJ The Role of PPARs in Lung Fibrosis PPAR Res 2007 2007 71323 17710235 10.1155/2007/71323 PMC1940051

[B18] Ward JE Tan X Peroxisome proliferator activated receptor ligands as regulators of airway inflammation and remodelling in chronic lung disease PPAR Res 2007 2007 14983 18000530 10.1155/2007/14983 PMC2065911

[B19] Ghosh AK Bhattacharyya S Lakos G Chen SJ Mori Y Varga J Disruption of transforming growth factor beta signaling and profibrotic responses in normal skin fibroblasts by peroxisome proliferator-activated receptor gamma Arthritis Rheum 2004 50 4 1305 1318 10.1002/art.20104 15077315

[B20] Langenbach SY Wheaton BJ Fernandes DJ Jones C Sutherland TE Wraith BC Harris T Schuliga MJ McLean C Stewart AG Resistance of fibrogenic responses to glucocorticoid and 2-methoxyestradiol in bleomycin-induced lung fibrosis in mice Can J Physiol Pharmacol 2007 85 7 727 738 10.1139/Y07-065 17823636

[B21] Huang JT Welch JS Ricote M Binder CJ Willson TM Kelly C Witztum JL Funk CD Conrad D Glass CK Interleukin-4-dependent production of PPAR-gamma ligands in macrophages by 12/15-lipoxygenase Nature 1999 400 6742 378 382 10.1038/22572 10432118

[B22] Allred CD Kilgore MW Selective activation of PPARgamma in breast, colon, and lung cancer cell lines Mol Cell Endocrinol 2005 235 1-2 21 29 10.1016/j.mce.2005.02.003 15866424

[B23] Allred CD Talbert DR Southard RC Wang X Kilgore MW PPARgamma1 as a molecular target of eicosapentaenoic acid in human colon cancer (HT-29) cells J Nutr 2008 138 2 250 256 18203887 10.1093/jn/138.2.250

[B24] Ward JE Gould H Harris T Bonacci JV Stewart AG PPARgamma ligands, 15-deoxy-Delta12,14-prostaglandin J2 and rosiglitazone regulate human cultured airway smooth muscle proliferation through different mechanisms Br J Pharmacol 2004 141 3 517 525 10.1038/sj.bjp.0705630 14718259 PMC1574213

[B25] Kim KK Wei Y Szekeres C Kugler MC Wolters PJ Hill ML Frank JA Brumwell AN Wheeler SE Kreidberg JA Epithelial cell alpha3beta1 integrin links beta-catenin and Smad signaling to promote myofibroblast formation and pulmonary fibrosis J Clin Invest 2009 119 1 213 224 10.1172/JCI38443C1 19104148 PMC2613463

[B26] Willis BC Borok Z TGF-beta-induced EMT: mechanisms and implications for fibrotic lung disease Am J Physiol Lung Cell Mol Physiol 2007 293 3 L525 534 10.1152/ajplung.00163.2007 17631612

[B27] Willis BC duBois RM Borok Z Epithelial origin of myofibroblasts during fibrosis in the lung Proc Am Thorac Soc 2006 3 4 377 382 10.1513/pats.200601-004TK 16738204 PMC2658689

[B28] Yamada M Kuwano K Maeyama T Hamada N Yoshimi M Nakanishi Y Kasper M Dual-immunohistochemistry provides little evidence for epithelial-mesenchymal transition in pulmonary fibrosis Histochem Cell Biol 2008 129 4 453 462 10.1007/s00418-008-0388-9 18236067

[B29] Foster KA Oster CG Mayer MM Avery ML Audus KL Characterization of the A549 cell line as a type II pulmonary epithelial cell model for drug metabolism Exp Cell Res 1998 243 2 359 366 10.1006/excr.1998.4172 9743595

[B30] Ando S Otani H Yagi Y Kawai K Araki H Fukuhara S Inagaki C Proteinase-activated receptor 4 stimulation-induced epithelial-mesenchymal transition in alveolar epithelial cells Respir Res 2007 8 31 10.1186/1465-9921-8-31 17433115 PMC1855055

[B31] Shintani Y Maeda M Chaika N Johnson KR Wheelock MJ Collagen I promotes epithelial-to-mesenchymal transition in lung cancer cells via transforming growth factor-beta signaling Am J Respir Cell Mol Biol 2008 38 1 95 104 10.1165/rcmb.2007-0071OC 17673689 PMC2176131

[B32] Illman SA Lehti K Keski-Oja J Lohi J Epilysin (MMP-28) induces TGF-beta mediated epithelial to mesenchymal transition in lung carcinoma cells J Cell Sci 2006 119 Pt 18 3856 3865 10.1242/jcs.03157 16940349

[B33] Chen L Necela BM Su W Yanagisawa M Anastasiadis PZ Fields AP Thompson EA Peroxisome proliferator-activated receptor gamma promotes epithelial to mesenchymal transformation by Rho GTPase-dependent activation of ERK1/2 J Biol Chem 2006 281 34 24575 24587 10.1074/jbc.M604147200 16815847

[B34] Hatta K Takeichi M Expression of N-cadherin adhesion molecules associated with early morphogenetic events in chick development Nature 1986 320 6061 447 449 10.1038/320447a0 3515198

[B35] Matsuyoshi N Imamura S Multiple cadherins are expressed in human fibroblasts Biochem Biophys Res Commun 1997 235 2 355 358 10.1006/bbrc.1997.6707 9199196

[B36] Hinz B Pittet P Smith-Clerc J Chaponnier C Meister JJ Myofibroblast development is characterized by specific cell-cell adherens junctions Mol Biol Cell 2004 15 9 4310 4320 10.1091/mbc.E04-05-0386 15240821 PMC515361

[B37] Shukla MN Rose JL Ray R Lathrop KL Ray A Ray P Hepatocyte Growth Factor Inhibits Epithelial to Myofibroblast Transition in Lung Cells Via Smad7 Am J Respir Cell Mol Biol 2008 18988920 10.1165/rcmb.2008-0217OC PMC2689916

[B38] Xu GP Li QQ Cao XX Chen Q Zhao ZH Diao ZQ Xu ZD The Effect of TGF-beta1 and SMAD7 gene transfer on the phenotypic changes of rat alveolar epithelial cells Cell Mol Biol Lett 2007 17457524 10.2478/s11658-007-0018-x PMC6275908

[B39] Chu YS Eder O Thomas WA Simcha I Pincet F Ben-Ze'ev A Perez E Thiery JP Dufour S Prototypical type I E-cadherin and type II cadherin-7 mediate very distinct adhesiveness through their extracellular domains J Biol Chem 2006 281 5 2901 2910 10.1074/jbc.M506185200 16253998

[B40] Islam S Carey TE Wolf GT Wheelock MJ Johnson KR Expression of N-cadherin by human squamous carcinoma cells induces a scattered fibroblastic phenotype with disrupted cell-cell adhesion J Cell Biol 1996 135 6 Pt 1 1643 1654 10.1083/jcb.135.6.1643 8978829 PMC2133960

[B41] Bonniaud P Margetts PJ Kolb M Haberberger T Kelly M Robertson J Gauldie J Adenoviral gene transfer of connective tissue growth factor in the lung induces transient fibrosis Am J Respir Crit Care Med 2003 168 7 770 778 10.1164/rccm.200210-1254OC 12816739

[B42] Birkedal-Hansen H Proteolytic remodeling of extracellular matrix Curr Opin Cell Biol 1995 7 5 728 735 10.1016/0955-0674(95)80116-2 8573349

[B43] Lenz O Elliot SJ Stetler-Stevenson WG Matrix metalloproteinases in renal development and disease J Am Soc Nephrol 2000 11 3 574 581 10703682 10.1681/ASN.V113574

[B44] Yang J Liu Y Dissection of key events in tubular epithelial to myofibroblast transition and its implications in renal interstitial fibrosis Am J Pathol 2001 159 4 1465 1475 11583974 10.1016/S0002-9440(10)62533-3 PMC1850509

[B45] Boes M Dake BL Booth BA Erondu NE Oh Y Hwa V Rosenfeld R Bar RS Connective tissue growth factor (IGFBP-rP2) expression and regulation in cultured bovine endothelial cells Endocrinology 1999 140 4 1575 1580 10.1210/en.140.4.1575 10098490

[B46] Lehmann JM Moore LB Smith-Oliver TA Wilkison WO Willson TM Kliewer SA An antidiabetic thiazolidinedione is a high affinity ligand for peroxisome proliferator-activated receptor gamma (PPAR gamma) J Biol Chem 1995 270 22 12953 12956 10.1074/jbc.270.22.12953 7768881

[B47] Willson TM Cobb JE Cowan DJ Wiethe RW Correa ID Prakash SR Beck KD Moore LB Kliewer SA Lehmann JM The structure-activity relationship between peroxisome proliferator-activated receptor gamma agonism and the antihyperglycemic activity of thiazolidinediones J Med Chem 1996 39 3 665 668 10.1021/jm950395a 8576907

[B48] Fu M Zhang J Zhu X Myles DE Willson TM Liu X Chen YE Peroxisome proliferator-activated receptor gamma inhibits transforming growth factor beta-induced connective tissue growth factor expression in human aortic smooth muscle cells by interfering with Smad3 J Biol Chem 2001 276 49 45888 45894 10.1074/jbc.M105490200 11590167

[B49] Saika S Yamanaka O Okada Y Miyamoto T Kitano A Flanders KC Ohnishi Y Nakajima Y Kao WW Ikeda K Effect of overexpression of PPARgamma on the healing process of corneal alkali burn in mice Am J Physiol Cell Physiol 2007 293 1 C75 86 10.1152/ajpcell.00332.2006 17625041

[B50] Ghosh A Bhattacharyya S Wei J Kim S Barak Y Mori Y Varga J Peroxisome proliferator-activated receptor-{gamma} abrogates Smad-dependent collagen stimulation by targeting the p300 transcriptional coactivator FASEB Journal 2009 19395477 10.1096/fj.08-128736 PMC2735362

[B51] Poncelet AC Schnaper HW Sp1 and Smad proteins cooperate to mediate transforming growth factor-beta 1-induced alpha 2(I) collagen expression in human glomerular mesangial cells J Biol Chem 2001 276 10 6983 6992 10.1074/jbc.M006442200 11114293

[B52] Necela BM Su W Thompson EA Peroxisome proliferator-activated receptor gamma down-regulates follistatin in intestinal epithelial cells through SP1 J Biol Chem 2008 283 44 29784 29794 10.1074/jbc.M804481200 18768463 PMC2662057

[B53] Wu M Melichian DS Chang E Warner-Blankenship M Ghosh AK Varga J Rosiglitazone abrogates bleomycin-induced scleroderma and blocks profibrotic responses through peroxisome proliferator-activated receptor-gamma Am J Pathol 2009 174 2 519 533 10.2353/ajpath.2009.080574 19147827 PMC2630560

[B54] Cho H Tai HH Thiazolidinediones as a novel class of NAD(+)-dependent 15-hydroxyprostaglandin dehydrogenase inhibitors Arch Biochem Biophys 2002 405 2 247 251 10.1016/S0003-9861(02)00352-1 12220539

[B55] Feinstein DL Spagnolo A Akar C Weinberg G Murphy P Gavrilyuk V Dello Russo C Receptor-independent actions of PPAR thiazolidinedione agonists: is mitochondrial function the key? Biochem Pharmacol 2005 70 2 177 188 10.1016/j.bcp.2005.03.033 15925327

